# Distribution of *HLA-B* Alleles and Haplotypes in Qatari: Recommendation for Establishing Pharmacogenomic Markers Screening for Drug Hypersensitivity

**DOI:** 10.3389/fphar.2022.891838

**Published:** 2022-08-08

**Authors:** Mohammed Dashti, Abdullah Al-Matrouk, Arshad Channanath, Prashantha Hebbar, Fahd Al-Mulla, Thangavel Alphonse Thanaraj

**Affiliations:** ^1^ Genetics and Bioinformatics Department, Dasman Diabetes Institute, Kuwait City, Kuwait; ^2^ Narcotic and Psychotropic Department, Ministry of Interior, Farwaniya, Kuwait

**Keywords:** HLA-B alleles, pharmacogenetics, drug hypersensitivity, HLA typing class I, Qatari population

## Abstract

Human leukocyte antigen (HLA) proteins are present at the cellular surface of antigen-presenting cells and play a crucial role in the adaptive immune response. Class I genes, specifically certain *HLA-B* alleles, are associated with adverse drug reactions (ADRs) and are used as pharmacogenetic markers. Although ADRs are a common causes of hospitalization and mortality, the data on the prevalence of *HLA-B* pharmacogenetics markers in Arab countries are scarce. In this study, we investigated the frequencies of major *HLA-B* pharmacogenomics markers in the Qatari population. Next-generation sequencing data from 1,098 Qatari individuals were employed for *HLA-B* typing using HLA-HD version 1.4.0 and IPD-IMGT/HLA database. In addition, *HLA-B* pharmacogenetics markers were obtained from the HLA Adverse Drug Reaction Database. In total, 469 major *HLA-B* pharmacogenetic markers were identified, with HLA-B*51:01 being the most frequent pharmacogenetic marker (26.67%) in the Qatari population. Moreover, HLA-B*51:01 is associated with phenytoin- and clindamycin-induced ADRs. The second most frequent pharmacogenetic marker was the HLA-B*58:01 allele (6.56%), which is associated with allopurinol-induced ADRs. The third most frequent pharmacogenetic marker was the HLA-B*44:03 allele, which is associated with phenytoin-induced ADRs. The establishment of a pharmacogenetics screening program in Qatar for cost effective interventions aimed at preventing drug-induced hypersensitivity can be aided by the highly prevalent *HLA-B* pharmacogenetic markers detected here.

## Introduction

Human leukocyte antigen (HLA) proteins are encoded by the *HLA* gene complex, which is located on the short arm of chromosome 6, and are inherited from both parents, one being paternal and the other being maternal (Choo, 2007). These molecules are present at the cellular surface of antigen-presenting cells and play a crucial role in the adaptive immune response. The *HLA* antigens are classified into three classes according to their coding gene locus, function, expression, and biochemical characteristics (classes I, II, and III). The classical *HLA* loci consist of class I molecules (HLA-A, -B, and -C); class II molecules, which are encoded by six main genes, namely, *HLA-DPA1*, *HLA-DPB1*, *HLA-DQA1*, *HLA-DQB1*, *HLA-DRA*, and *HLA-DRB1*; and class III antigens, which are encoded by the *HLA-Bf*, *C2*, and *C4A* loci (Ulvestad et al., 1994; Howell et al., 2010). Class I molecules present peptides derived from intracellular proteins to cytotoxic T cells (CD8^+^), whereas class II peptides present internalized exogenous proteins to T helper cells (CD4^+^) (Reviewed by Dendrou et al., 2018).

The *HLA* genes are the most polymorphic genetic region in the human genome, as they need to present a huge variety of peptides. Different populations and ethnic groups were shown to encode different *HLA* alleles. In addition, there is a cumulative body of evidence showing that certain *HLA-B* alleles variants can be used as pharmacogenomic markers to predict adverse drug reactions (ADRs) and hypersensitivities, as several drugs were shown to induce immune hypersensitivity responses through interactions with *HLA-B* proteins (Jung et al., 2018). Such allele variants, which are significantly associated with responses to specific types of drugs, are referred to as pharmacogenetic markers. It has been observed that pharmacogenetic markers are usually drug-specific, phenotype-specific, and population-specific markers (Supplementary Table S1).

ADRs were defined by Edwards and Aronson (2000) as “an appreciably harmful or unpleasant” reaction resulting from an intervention related to the use of a medicinal product, which predicts hazard from future administration and warrants prevention/specific treatment, alteration of the dosage regimen, or withdrawal of the product. ADRs are considered a major health issue as they are a common cause of hospitalization and mortality (Naisbitt et al., 2003; Gomes and Demoly, 2005). Traditionally, ADRs have been classified into two major types, i.e., Type A or augmented reaction, and Type B or bizarre reaction. Type A is dose dependent and is predicted based on the pharmacology of the drug, whereas Type B is idiosyncratic and is unpredicted based on pharmacology, a it is primarily determined by host genetics. Type B, although less frequent (representing approximately 10%–15% of all ADR cases) than Type A, is relatively more severe (Pirmohamed, 2010). Hypersensitivity drug reactions, which are a Type B reaction, have clinical manifestations, with cutaneous adverse drug reactions (CADRs) being the most common. Some of the CADRs can be classified as severe cutaneous adverse reactions (SCARs), including Stevens–Johnson syndrome (SJS), toxic epidermal necrolysis (TEN), drug reactions with eosinophilia and systemic symptoms (DRESS), drug-induced hypersensitivity syndrome, or hypersensitivity syndrome (HSS) (Sukasem et al., 2014). These SCARs (with no exception) are characterized by considerable rates of morbidity and mortality. However, each SCAR has its own characteristic cutaneous presentations, causative drugs, clinical courses, pathomechanisms, and possible treatment modalities (Pichler et al., 2011; Wei et al., 2012).

A previous study conducted on the Kuwaiti population showed that B*50:01g is one of the most common groups of *HLA-B* alleles, whereas in Saudi Arabia, B*51:01:01G (19.0%), and B*50:01:01G (12.3%) are the most common groups of *HLA-B* alleles (Ameen et al., 2020; Jawdat et al., 2020). Upper G denotes to merged group of exons 2 and 3 for HLA class I and exon 2 for HLA class II alleles with identical nucleotide sequences (Marsh et al., 2010). Lower g denotes to merged group of alleles that differ in nonsynonymous mutations outside the relevant exons, and synonymous mutations inside or outside the relevant exons (Schmidt et al., 2009; Schaefer et al., 2017). Moreover, in other Arab populations, the B*35 group of alleles was detected at high frequencies among Omanis (15.3%), Jordanians (14%), and Arab Emirati (11.1%) (Sanchez-Velasco et al., 2001; Elbjeirami et al., 2013; Albalushi et al., 2014). B*51 is another group of alleles that has been found at high frequencies among Saudis (19.3%), Omanis (14.7%), and Arab Emiratis (15.6%) (Sanchez-Velasco et al., 2001; Albalushi et al., 2014). In addition, B*50 is also a frequent allele group in most Arabs, including Saudis (18.8%) and Libyans (16.1%), together with B*08 and B*44 among the Tunisian Berbers of Zrawa (32.8%), with the latter being the highest frequency recorded worldwide (Sanchez-Velasco et al., 2001; Hajjej et al., 2011). In contrast, the B*37, 42, 46, 47, 48, 54, 59, 67, and 78 allele groups are extremely rare or virtually absent in all Arab populations (Hajjej et al., 2011). Among Syrians, the frequency of the B*35 allele group is 18.6%, whereas that of B*44 is 7.6% and that of B*51 is 8.1% (Ikhtiar et al., 2018). Table 1 shows the top 10 most frequent HLA-B alleles in the Arab population.

**TABLE 1:** Top 10 HLA-B frequent alleles in the Arab population.

Kuwait		Saudi		Omani		Jordan		Tunisia		Syria	
HLA-B	AF (%)	HLA-B	AF (%)	HLA-B	AF(%)	HLA-B	AF(%)	*HLA-B*	AF(%)	HLA-B	AF (%)
B*50:01g	12.02	B*51:01:01G	19.0	B*35	15.3	B*35	14.9	B*51:01	6.8	B*35	18.6
B*51:01g	10.49	B*50:01:01G	12.4	B*51	14.7	B*51	10.3	B*08:01	6.7	B*51	8.1
B*08:01g	7.23	B*08:01:01G	6.9	B*08	9.3	B*50	6.4	B*07:02	4.4	B*44	7.6
B*52:01g	4.20	B*07:02:01G	5.0	B*58	9.1	B*49	6.2	B*52:01:01	2.0	B*14	6.2
B*41:01	4.03	B*53:01:01G	3.9	B*40	6.4	B*41	5.8	B*55:01	1.5	B*52	7.1
B*35:01g	3.74	B*41:01	3.4	B*52	6.0	B*44	5.6	B*50:04	1.7	B*49	5.7
B*07:02g	3.70	B*58:01:01G	3.4	B*15	6.0	B*18	5.3	B*58:01	2.0	B*38	5.7
B*18:01g	3.61	B*35:01:01G	2.8	B*18	4.2	B*52	4.9	B*53:01	1.1	B*08	4.8
B*40:06	3.61	B*18:01:01G	2.7	B*50	4.2	B*15	4.7	B*57:03	0.9	B*18	4.8
B*58:01g	3.19	B*49:01:01G	2.5	B*07	3.1	B*38	4.7	B*51:08	0.7	B* 4.3	4.3
Ameen et al. (2020)		Jawdat et al. (2020)		Albalushi et al. (2014)		Elbjeirami et al. (2013)		Hajjej et al. (2011)		Ikhtiar et al. (2018)	
References											

Unfortunately, the published data connecting the frequencies of *HLA-B* haplotypes and alleles with pharmacogenetics in the Arab countries are scarce. Providing such data would lead to a better understanding of the pharmacogenomics of the Arab population and would further shed light on associations between ADRs and *HLA*. Thus, the current study will highlight this issue.

## Materials and Methods

### Ethics Statement

The current study was reviewed and approved by the institutional Ethical Review Committee at the Dasman Diabetes Institute, Kuwait in accordance with the declaration of Helsinki. The genomic data of Qatari individuals used in this study are publicly available from the National Center for Biotechnology Information Sequence Read Archive. Informed consent was obtained from the recruited participants of the original studies (Fakhro et al., 2016; Rodriguez-Flores et al., 2016) under protocols approved by the Institutional Review Boards of Hamad Medical Corporation and Weill Cornell Medical College in Qatar.

### Study Samples

The whole exome sequencing data of individuals living in Qatar (Fakhro et al., 2016; Rodriguez-Flores et al., 2016), as sequenced using Agilent SureSelect Human All Exon V5 and V4 kits (Agilent Technologies Inc., United States) on an Illumina HiSeq platform, are publicly available from the National Center for Biotechnology Information Sequence Read Archive, with the following accessions: SRP060765, SRP061943, and SRP061463. Only 1,000 exomes of native Qatari individuals were considered for this study as they were sequenced with the Agilent V5 kit (Agilent Technologies Inc., United States). Furthermore, 98 native Qatari individuals, whose whole genome sequence data were available, were also used in our study. In total, 1,098 individuals were enrolled in this study: 475 males (43.57%) and 623 females (57.15%) with an average age of 50 years. Three samples were excluded from this study as they failed to meet the quality control threshold resulting in untyped *HLA-B* alleles, whereas *HLA-B* loci were successfully sequenced in the samples of the remaining 1,095 individuals.

### Quality Control

Furthermore, we downloaded the whole genome sequencing data from an additional eight individuals (common with whole exome samples, sharing the same sample identification number) available from the same Qatari studies (Fakhro et al., 2016; Rodriguez-Flores et al., 2016) and were used to validate the *HLA* typing from whole exome data.

### *HLA-B* Typing

The FastQ files of 1,098 Qatari individuals were used as input for the HLA-HD tool version 1.4.0 (Kawaguchi et al., 2017), to determine the HLA alleles by mapping reads to a comprehensive reference panel from the IPD-IMGT/HLA database (Robinson et al., 2015) version 3.46 (2021-October) build 2d19adf. The companion website to the official repository is hla.alleles.org. The data are also accessible at https://www.ebi.ac.uk/ipd/imgt/hla/licence/.

### *HLA-B* Pharmacogenomic Markers

Major *HLA-B* pharmacogenetic markers were obtained from the HLA Adverse Drug Reaction Database (HLA-ADR) website (http://www.allelefrequencies.net/).

### Statistical Analysis

*HLA-B* allele frequency (AF) calculations were carried out by direct counting, followed by dividing the total number of occurrences of the specific allele by the total number of B alleles in a population. *HLA-B* AF are assessed for deviation from Hardy–Weinberg equilibrium (HWE) using the R software (https://www.R-project.org/) version 3.6.2.

## Results

### *HLA-B* Allelic Frequencies

After demonstrating the effectiveness of *HLA-B* typing using next-generation sequencing (NGS) data (Supplementary Table S2), the frequency of the 107 different HLA-B alleles identified in the 1,098 Qatari individuals are listed in Supplementary Table S3 and Table 2 (*n* > 1). The most frequent *HLA-B* alleles were B*50:01 (18.21%), B*51:01 (17.35%), B*08:01 (7.24%), B*07:02 (4.64%), B*40:06 (4.37%), and B*58:01 (3.42%). *HLA-B* AF did not significantly deviate from HWE.

**TABLE 2:** *HLA-B* alleles (*n* > 1) frequency in Qatari population.

*HLA-B* alleles	No. of alleles	AF (%)	Estimated genotype	No. of genotypes	Genotype frequency (%)	HWE *p*-value
B*50:01	400	18.21	36.43	51	4.64	0.13
B*51:01	381	17.35	33.05	48	4.37	0.11
B*08:01	159	7.24	5.76	9	0.82	0.6
B*07:02	102	4.64	2.37	4	0.36	0.68
B*40:06	96	4.37	2.1	6	0.55	0.29
B*58:01	75	3.42	1.28	3	0.27	0.62
B*18:01	62	2.82	0.88	3	0.27	0.62
B*49:01	62	2.82	0.88	4	0.36	0.37
B*53:01	60	2.73	0.82	1	0.09	0.48
B*35:01	56	2.55	0.71	2	0.18	1
B*52:01	47	2.14	0.5	1	0.09	1
B*35:03	39	1.78	0.35	0	0	1
B*35:08	37	1.68	0.31	1	0.09	1
B*35:02	35	1.59	0.28	0	0	1
B*14:02	34	1.55	0.26	0	0	1
B*55:01	34	1.55	0.26	1	0.09	1
B*41:01	33	1.5	0.25	0	0	1
B*44:03	31	1.41	0.22	0	0	1
B*15:17	30	1.37	0.2	0	0	1
B*57:01	30	1.37	0.2	0	0	1
B*37:01	29	1.32	0.19	1	0.09	1
B*15:03	20	0.91	0.09	1	0.09	1
B*15:10	19	0.87	0.08	2	0.18	1
B*38:01	19	0.87	0.08	3	0.27	1
B*45:01	17	0.77	0.07	0	0	1
B*73:01	17	0.77	0.07	0	0	1
B*39:10	16	0.73	0.06	0	0	1
B*42:01	16	0.73	0.06	1	0.09	1
B*51:08	16	0.73	0.06	0	0	1
B*13:02	15	0.68	0.05	0	0	1
B*44:02	12	0.55	0.03	1	0.09	1
B*07:05	11	0.5	0.03	0	0	1
B*27:02	11	0.5	0.03	1	0.09	1
B*40:01	10	0.46	0.02	0	0	1
B*39:01	9	0.41	0.02	0	0	1
B*81:01	8	0.36	0.01	1	0.09	1
B*15:220	7	0.32	0.01	0	0	1
B*57:03	7	0.32	0.01	0	0	1
B*58:02	7	0.32	0.01	0	0	1
B*14:01	6	0.27	0.01	0	0	1
B*27:05	6	0.27	0.01	0	0	1
B*39:24	6	0.27	0.01	0	0	1
B*50:57	6	0.27	0.01	0	0	1
B*51:02	6	0.27	0.01	0	0	1
B*07:06	4	0.18	0	0	0	1
B*07:381	4	0.18	0	0	0	1
B*15:01	4	0.18	0	0	0	1
B*15:08	3	0.14	0	0	0	1
B*35:05	3	0.14	0	0	0	1
B*40:02	3	0.14	0	0	0	1
B*40:449	3	0.14	0	0	0	1
B*47:03	3	0.14	0	0	0	1
B*51:230	3	0.14	0	0	0	1
B*57:02	3	0.14	0	0	0	1
B*13:01	2	0.09	0	0	0	1
B*15:02	2	0.09	0	0	0	1
B*15:18	2	0.09	0	0	0	1
B*15:31	2	0.09	0	0	0	1
B*18:43	2	0.09	0	0	0	1
B*27:07	2	0.09	0	0	0	1
B*41:02	2	0.09	0	0	0	1
B*42:02	2	0.09	0	0	0	1
B*44:302	2	0.09	0	0	0	1
B*44:464	2	0.09	0	0	0	1
B*51:143	2	0.09	0	0	0	1

### *HLA-B* Genotype Frequencies

428 different *HLA-B* genotypes were identified in the 1,098 Qatari individuals. The top 10 most frequent *HLA-B* genotypes are listed in Table 3. The B*50:01 + B*51:01 genotype emerged as the most common genotype at a frequency of 8.56% in the Qatari population. The remainder of the frequent genotypes exhibited a frequency of <5% in the Qatari population.

**TABLE 3:** Top 10 *HLA-B* genotypes frequency in Qatari population.

*HLA-B* genotypes	No of individuals	Frequency (%)
B*50:01 + B*51:01	94	8.56
B*50:01 + B*50:01	51	4.64
B*51:01 + B*51:01	48	4.37
B*08:01 + B*50:01	29	2.64
B*08:01 + B*51:01	21	1.91
B*07:02 + B*50:01	20	1.82
B*07:02 + B*51:01	17	1.55
B*49:01 + B*51:01	14	1.28
B*49:01 + B*50:01	13	1.18
B*51:01 + B*53:01	12	1.09

### Frequency of Major *HLA-B* Pharmacogenetics Markers in the Qatari Population

In total, 469 major *HLA-B* pharmacological markers were identified when we screened the *HLA-B* markers from the 1,098 Qatari individuals (Table 3). The most frequent pharmacogenetic marker in the Qatari population was HLA-B*51:01 (26.67%), with 329 individuals carrying this marker, which is associated with phenytoin- and clindamycin-induced ADRs. Among them, 55% were women and 45% were men. In contrast, only 48 Qatari individuals were shown to carry the homozygous HLA-B*51:01 genotype, with the remainder of the cohort carrying heterozygous genotypes. The second most frequent pharmacogenetic marker was the HLA-B*58:01 allele, which was carried by 72 Qatari individuals (6.56%) and is associated with allopurinol-induced CADRs; 58% of the individuals were women and 42% were men. Furthermore, only three individuals carried the homozygous HLA-B*58:01 genotype and 69 individuals carried the heterozygous B*58:01 genotype. The third most frequent pharmacogenetic marker was the HLA-B*44:03 allele, which is associated with phenytoin-induced ADRs, with 61% of the individuals who carried this allele being women and 39% being men. Moreover, none of the Qatari individuals carried the homozygous HLA-B*44:03 genotype. The fourth most frequent pharmacogenetic marker was the HLA-B*57:01 allele (2.72%), which is involved in abacavir-induced hypersensitivity syndrome (AHS); 73% of the carriers were women and 27% were men. Finally, none of the Qatari individuals carried the homozygous HLA-B*57:01 genotype.

## Discussion

The Middle East constitutes to be a historic intersection of human civilizations and migrations. Qatar exhibits extensive diversity as well as genetic ancestries representing the main founding Arab genealogical lineages of Qahtanite (Peninsular Arabs) and Adnanite (General Arabs and West Eurasian Arabs) (Razali et al., 2021). A principal components analysis on genome-wide genotype data from Qatar revealed three clear clusters of genotypes, the proximity of which to other human population samples is consistent with an Arabian origin, a more eastern or Persian origin, and individuals with African admixture (Hunter-Zinck et al., 2010).

In the current study, we employed the publicly available NGS data for the Qatari population, with the majority being from the whole exome of 1,098 individuals, to report the *HLA-B* locus diversity in the Qatari population for the first time.

Several previous studies were conducted on the *HLA-B* AF, covering different GCC countries, including Oman, Saudi Arabia, Kuwait, and the United Arab Emirates (Williams et al., 2001; Hajeer et al., 2013; Ameen et al., 2020; Jawdat et al., 2020). In addition, similar studies were conducted in other Arabic countries, including Libya, Tunisia, Syria, and Jordan (Elbjeirami et al., 2013; Hajjej et al., 2015; Jazairi et al., 2016; Ikhtiar et al., 2018). However, there is a lack of data regarding AF in Qatar compared with the remainder of the Arabic countries (Table 1). Thus, our study was the first to shed light on *HLA* diversity in this population.

The analysis of the obtained NGS data has demonstrated a good coverage across the major histocompatibility complex (MHC) region, which resulted in high-resolution *HLA-B* typing (three fields). Therefore, this can be used to discriminate between samples (Supplementary Table S2) specifically from whole exome data.

In our study, we identified 107 allele types from the delineated NGS data that were generated from 1,098 Qatari individuals; furthermore, by analyzing the frequencies of those alleles we showed that they passed the tests for HWE (Table 4). The most frequently observed *HLA-B* alleles were B*50:01 (18.21%), B*51:01 (17.35%), B*08:01 (7.24%), and B*07:02 (4.64%). These most frequent *HLA-B* alleles and genotype were very similar to the findings reported for the Kuwaiti (Ameen et al., 2020), Saudi Arabian (Hajeer et al., 2013; Jawdat et al., 2020), and Omani (Williams et al., 2001) populations, and exhibited comparable frequencies to those of the surrounding Arab populations, such as the Jordanian (Elbjeirami et al., 2013), Tunisian (Hajjej et al., 2015), and Syrian (Jazairi et al., 2016; Ikhtiar et al., 2018) populations. This was not the case regarding *HLA-B* allele studies in other populations from Thailand (Puangpetch et al., 2015), China (Middleton et al., 2004), Singapore (Williams et al., 2001), Malaysia (Jinam et al., 2010), and European American (Creary et al., 2019), which are not close geographically. The sharing of the most frequent alleles among GCC countries compared with other Arab countries could be mainly attributed to a similar gene pool; the close geographical location of the GCC countries; and the sharing of similar culture, language, religion, history, and ancestors.

**TABLE 4:** Genotype frequency of major *HLA-B* pharmacogenetics markers in Qatari population.

Pharmacogenetic marker	Genotype	Individuals	Frequency (%)
HLA-B*13:01 *n* = 2	B*13:01 + B*51:01	2	0.18
HLA-B*15:02 *n* = 2	B*15:02 + B*38:01	1	0.09
	B*15:02 + B*51:08	1	0.09
HLA-B*35:05 *n* = 3	B*35:05 + B*08:01	1	0.09
	B*35:05 + B*40:06	1	0.09
	B*35:05 + B*58:01	1	0.09
HLA-B*44:03 *n* = 31	B*44:03 + B*50:01	4	0.36
	B*44:03 + B*08:01	3	0.27
	B*44:03 + B*35:01	3	0.27
	B*44:03 + B*40:01	2	0.18
	B*44:03 + B*51:01	2	0.18
	B*44:03 + B*57:01	2	0.18
	B*44:03 + B*07:02	1	0.09
	B*44:03 + B*15:03	1	0.09
	B*44:03 + B*15:10	1	0.09
	B*44:03 + B*15:17	1	0.09
	B*44:03 + B*15:31	1	0.09
	B*44:03 + B*35:03	1	0.09
	B*44:03 + B*35:08	1	0.09
	B*44:03 + B*38:01	1	0.09
	B*44:03 + B*39:01	1	0.09
	B*44:03 + B*40:06	1	0.09
	B*44:03 + B*44:02	1	0.09
	B*44:03 + B*49:01	1	0.09
	B*44:03 + B*50:57	1	0.09
	B*44:03 + B*52:01	1	0.09
	B*44:03 + B*55:01	1	0.09
HLA-B*51:01 *n* = 329	B*51:01 + B*50:01	94	8.56
	B*51:01 + B*51:01	48	4.37
	B*51:01 + B*08:01	21	1.91
	B*51:01 + B*07:02	17	1.55
	B*51:01 + B*49:01	14	1.28
	B*51:01 + B*53:01	12	1.09
	B*51:01 + B*58:01	11	1
	B*51:01 + B*18:01	9	0.82
	B*51:01 + B*40:06	8	0.73
	B*51:01 + B*15:17	7	0.64
	B*51:01 + B*37:01	6	0.55
	B*51:01 + B*35:03	6	0.55
	B*51:01 + B*35:02	6	0.55
	B*51:01 + B*73:01	5	0.46
	B*51:01 + B*52:01	4	0.36
	B*51:01 + B*35:01	4	0.36
	B*51:01 + B*14:02	3	0.36
	B*51:01 + B*81:01	3	0.27
	B*51:01 + B*51:02	3	0.27
	B*51:01 + B*44:02	3	0.27
	B*51:01 + B*41:01	3	0.27
	B*51:01 + B*13:02	3	0.27
	B*51:01 + B*57:01	2	0.18
	B*51:01 + B*51:143	2	0.18
	B*51:01 + B*44:03	2	0.18
	B*51:01 + B*42:01	2	0.18
	B*51:01 + B*39:10	2	0.18
	B*51:01 + B*27:05	2	0.18
	B*51:01 + B*15:220	2	0.18
	B*51:01 + B*15:10	2	0.18
	B*51:01 + B*15:03	2	0.18
	B*51:01 + B*15:01	2	0.18
	B*51:01 + B*13:01	2	0.18
	B*51:01 + B*07:06	2	0.18
	B*51:01 + B*07:05	2	0.18
	B*51:01 + B*07:303	1	0.09
	B*51:01 + B*08:170	1	0.09
	B*51:01 + B*15:151	1	0.09
	B*51:01 + B*15:31	1	0.09
	B*51:01 + B*27:02	1	0.09
	B*51:01 + B*35:08	1	0.09
	B*51:01 + B*38:163	1	0.09
	B*51:01 + B*39:01	1	0.09
	B*51:01 + B*39:24	1	0.09
	B*51:01 + B*40:449	1	0.09
	B*51:01 + B*51:08	1	0.09
	B*51:01 + B*55:01	1	0.09
	B*51:01 + B*57:03	1	0.09
HLA-B*57:01 *n* = 30	B*57:01 + B*07:02	5	0.46
	B*57:01 + B*50:01	5	0.46
	B*57:01 + B*14:02	4	0.36
	B*57:01 + B*40:06	3	0.27
	B*57:01 + B*44:03	2	0.18
	B*57:01 + B*51:01	2	0.18
	B*57:01 + B*08:01	1	0.09
	B*57:01 + B*15:18	1	0.09
	B*57:01 + B*35:01	1	0.09
	B*57:01 + B*35:03	1	0.09
	B*57:01 + B*35:08	1	0.09
	B*57:01 + B*37:01	1	0.09
	B*57:01 + B*38:01	1	0.09
	B*57:01 + B*41:35	1	0.09
	B*57:01 + B*45:01	1	0.09
HLA-B*58:01 *n* = 72	B*58:01 + B*51:01	11	1
	B*58:01 + B*40:06	8	0.73
	B*58:01 + B*50:01	6	0.55
	B*58:01 + B*07:02	4	0.36
	B*58:01 + B*53:01	4	0.36
	B*58:01 + B*35:03	3	0.27
	B*58:01 + B*52:01	3	0.27
	B*58:01 + B*58:01	3	0.27
	B*58:01 + B*08:01	2	0.18
	B*58:01 + B*15:10	2	0.18
	B*58:01 + B*15:17	2	0.18
	B*58:01 + B*45:01	2	0.18
	B*58:01 + B*49:01	2	0.18
	B*58:01 + B*55:01	2	0.18
	B*58:01 + B*57:03	2	0.18
	B*58:01 + B*14:01	1	0.09
	B*58:01 + B*14:02	1	0.09
	B*58:01 + B*15:220	1	0.09
	B*58:01 + B*18:01	1	0.09
	B*58:01 + B*27:02	1	0.09
	B*58:01 + B*35:01	1	0.09
	B*58:01 + B*35:05	1	0.09
	B*58:01 + B*37:01	1	0.09
	B*58:01 + B*39:10	1	0.09
	B*58:01 + B*39:24	1	0.09
	B*58:01 + B*40:01	1	0.09
	B*58:01 + B*42:01	1	0.09
	B*58:01 + B*51:08	1	0.09
	B*58:01 + B*57:02	1	0.09
	B*58:01 + B*57:38	1	0.09
	B*58:01 + B*73:01	1	0.09
Total		469	42.56

In addition, to the best of our knowledge, none of the previous studies of *HLA-B* AF in Arab countries (Williams et al., 2001; Elbjeirami et al., 2013; Hajjej et al., 2015; Ikhtiar et al., 2018; Ameen et al., 2020; Jawdat et al., 2020) investigated or reported pharmacogenomic data pertaining to genetic markers for genetic disorders, such as drug hypersensitivity. Thus, our study was the first of its kind to investigate and report genetic markers that were associated with ADRs. Moreover, obtaining such data by screening patients for *HLA-B* alleles is very useful, as it is a cost effective intervention for the prevention of ADRs.

Our data (Table 3) showed that HLA-B*51:01 was the most prevalent pharmacogenetic marker allele in the studied Qatari population. In the Han Chinese population, this genetic marker has been shown to be strongly associated with CADRs related to clindamycin, which is an antibiotic that is used for the treatment of several bacterial infections, including bone or joint infections, pelvic inflammatory disease, strep throat, pneumonia, middle ear infections, and endocarditis (Guay, 2007; Yang et al., 2017). Moreover, in the Thai population, it is also significantly associated with SCARs (such as SJS/TEN and DRESS) related to phenytoin (PHT), which is sold under the brand name Dilantin among others and is an anti-seizure medication that is useful for the prevention of tonic–clonic seizures (also known as grand mal seizures) and focal seizures (Tassaneeyakul et al., 2016). In addition, a very recent study demonstrated the association between HLA-B*51:01, HLA-B*55:01, and CYP2C9*3 and phenytoin-induced CADRs in the South Indian Tamil population (John et al., 2021). Another phenytoin-induced ADR allele is HLA-B*15:02, which also has been associated with other antiepileptic drugs, such as carbamazepine, in populations with Asian ancestry (Sukasem et al., 2018) and in the Spanish population (Ramirez et al., 2017); however, it had a rare prevalence in our study.

The HLA-B*57:01 and HLA-B*35:05 alleles, which have been found to be associated with antiviral-drug-induced hypersensitivity, were detected in 30 and 3 Qatari individuals, respectively. The HLA-B*57:01 allele was found to be associated with abacavir in Caucasians (Mallal et al., 2008), Western Australians (Mallal et al., 2002), and other populations (Hetherington et al., 2002; Hughes et al., 2004; Martin et al., 2004; Phillips et al., 2005). Abacavir (ABC) is an antiretroviral drug of the nucleoside reverse transcriptase inhibitor class, which is commonly combined with antiretroviral therapy for treating human immunodeficiency virus (HIV) infection. Abacavir acts as a potent guanosine nucleoside inhibitor of reverse transcriptase. However, approximately 5%–8% of patients experience a hypersensitivity reaction within the first 6 weeks of treatment, which can be severe and potentially life threatening (Ma et al., 2010). The other allele that has been shown to be associated with antiviral-drug-induced hypersensitivity is HLA-B*35:05, which was shown to be associated with nevirapine in the Thai population (Chantarangsu et al., 2009). Nevirapine (NVP) is sold under the brand name of Viramune and is used to treat and prevent HIV infection, specifically HIV-1. It is a non-nucleoside reverse transcriptase inhibitor that works by blocking the function of reverse transcriptase. The most common adverse effect of nevirapine is the development of a mild or moderate rash (13%). SCARs, including SJS/TEN and hypersensitivity, have been observed in 1.5% of patients (Pawar et al., 2015).

Moreover, our study showed that the HLA-B*58:01 allele had a high prevalence (72 individuals) in the Qatari population. This allele was shown to be associated with allopurinol-induced SJS/TEN in Asian populations, such as the Taiwanese (Ko et al., 2015), Korean (Kang et al., 2011), Japanese (Kaniwa et al., 2008), Thai (Tassaneeyakul et al., 2009), and some European (such as the Portuguese) (Goncalo et al., 2013) populations. This medication is used to decrease the elevated blood uric acid levels triggered by chemotherapy and to prevent gout and specific types of kidney stones (Jung et al., 2015).

The HLA-B*44:03 allele, which was carried by 2.8% of Qataris in our study, has been associated with cold-medicine-induced SJS/TEN in the Brazilian (Wakamatsu et al., 2015) and Japanese (Ueta et al., 2014) populations. Cold medicines include non-steroidal anti-inflammatory drugs and multi-ingredient cold medications. In another study, Park et al. (2016) stated that HLA-B*44:03 may be associated with lamotrigine-induced SJS/TEN among Koreans. Lamotrigine, which is sold under the brand name of lamictal, is used to treat epilepsy, including focal seizures, tonic–clonic seizures, and seizures in Lennox–Gastaut syndrome, and to prevent the recurrence of depressive–manic episodes in patients with bipolar disorder (Lorberg et al., 2009).

The HLA-B*13:01 allele was observed at an extremely low frequency in our study (two Qatari individuals) and was shown by others to be associated with Dapsone-induced SJS, TEN, and DRESS in Asian populations (Zhang et al., 2013; Tempark et al., 2017). Dapsone is used for the treatment of infection and inflammation, including leprosy, *Pneumocystis jiroveci* pneumonia, or *Toxoplasma gondii* encephalitis, in HIV prophylaxis, neutrophilic dermatoses, dermatitis herpetiformis, and autoimmune bullous disease (Tangamornsuksan and Lohitnavy, 2018). Dapsone hypersensitivity syndrome (DHS), or dapsone-induced hypersensitivity reactions, is a life threatening drug reaction that usually manifests itself between the 4th and 6th weeks after the initiation of treatment. Approximately 0.5%–3.6% of patients treated with dapsone have been reported to develop DHS, with a mortality rate of 9.9% (Fan et al., 2017; Tangamornsuksan and Lohitnavy, 2018).

The current study had several limitations, one of which was the absence of association studies between *HLA-B* alleles and medical drugs in the Middle East. As a result, some of the pharmacogenetic markers discussed in our study might be ethnicity specific based on the genetic background. For instance, in the Chinese (Chung et al., 2004), Thai (Tassaneeyakul et al., 2010), and European (Lonjou et al., 2008) populations, the association between the HLA-B*15:02 allele and carbamazepine-induced ADRs was significant, which was not the case in the Korean population (Kim et al., 2011). Although the NGS-based *HLA* typing has good resolution and we used duplicate samples (whole genome and whole exome) that showed consistent results, and despite the high sensitivity and specificity of the bioinformatics tools used here (Kawaguchi et al., 2017; Liu et al., 2021), the high variability of the MHC region can render NGS-based *HLA* typing prone to accuracy issues, as demonstrated in our study (Supplementary Table S2) and other studies (Major et al., 2013; Wittig et al., 2015; Larjo et al., 2017).

## Conclusion

In the current study, we observed similarities in the *HLA-B* alleles and genotypes in the Qatari population compared with other GCC countries. The prevalent alleles were also found to be associated with different drug-induced hypersensitivities in many other populations. Thus, we recommend performing a selected drug testing for some of the individuals that have been screened for *HLA-B* pharmacogenetic markers in a controlled clinical setting for the populations of the GCC countries. This is because such studies would be considered as a cost effective intervention for the prevention of drug-induced hypersensitivity.

## Data Availability

The datasets presented in this study can be found in online repositories. The names of the repository/repositories and accession number(s) can be found in the article/Supplementary Material.
