# Supplementary material for: Distribution of HLA-B Alleles and Haplotypes in Qatari: Recommendation for Establishing Pharmacogenomic Markers Screening for Drug Hypersensitivity
Source: Front Pharmacol. 2022 Aug 8;13:891838. doi: 10.3389/fphar.2022.891838 (PMC9393242; doi:10.3389/fphar.2022.891838)
Supplement: Supplementary file 4 [file DataSheet1.docx]

**Supplementary information**

1. Quality control results

A.1 Coverage of NGS data

Genome Analysis Tool Kit (GATK) version v3.8-1-0 (McKenna et al., 2010) was used to calculated average coverage after aligning reads from Qatari individuals that were sequenced by whole genome and exome to human assembly GRCh37 via Burrows-Wheeler Aligner using default parameters (Li and Durbin, 2009) then converted into binary format (BAM) with SAMtools (Li et al., 2009).

The genome region that was used to estimate the average coverage for all exome samples on exonic MHC region (chr6:28,477,797-33,448,354) was 64X. Whereas all whole genome Qatari samples calculated average coverage for the whole MHC region was 38X. A comparison between average coverage of 8 Qatari individuals who were sequenced by both genome and exome technologies are listed in supplementary Table S2.

A.2 Comparison of *HLA-B* genotype of same Qatari individuals using whole genome and exome NGS data

When comparing duplicates runs, 6 out of 8 duplicates samples have the same exact *HLA-B* (3 fields resolution) alleles and genotypes (see Supplementary table S2). One sample (DGMQ-31714) had the same allele group but not the same protein group which might be due to coverage issue. Another sample (DGMQ-31108) has significantly different *HLA-B* alleles. Nonetheless, using the same NGS pipeline to estimate the coverage to identify variants, a total of 1,072 variants within MHC region called from that individual using whole genome sequenced (SRR2098177) and whole exome sequenced (SRR5264032). The results shows that 131 variants are unique to either sequencing technology which may suggest that these supposedly duplicate runs are not from the same individual.

References:

Li, H., and Durbin, R. (2009). Fast and Accurate Short Read Alignment with Burrows-Wheeler Transform. *Bioinformatics* 25, 1754–1760. d[oi:10.1093/](https://doi.org/10.1093/bioinformatics/btp324) [bioinformatics/btp324](https://doi.org/10.1093/bioinformatics/btp324)

Li, H., Handsaker, B., Wysoker, A., Fennell, T., Ruan, J., Homer, N., et al. (2009). The Sequence Alignment/Map Format and SAMtools. *Bioinformatics* 25, 2078–2079. doi:10.1093[/bioinformatics/btp352](https://doi.org/10.1093/bioinformatics/btp352)

McKenna, A., Hanna, M., Banks, E., Sivachenko, A., Cibulskis, K., Kernytsky, A., et al. (2010). The Genome Analysis Toolkit: A MapReduce Framework for Analyzing Next-Generation DNA Sequencing Data. *Genome Res.* 20, 1297–1303. doi:10.1101[/gr.107524.110](https://doi.org/10.1101/gr.107524.110)
